# Randomized controlled clinical trial of a combination therapy of vildagliptin plus an α-glucosidase inhibitor for patients with type II diabetes mellitus

**DOI:** 10.3892/etm.2014.1637

**Published:** 2014-03-27

**Authors:** YONG SU, YA-LI SU, LI-FANG LV, LI-MIN WANG, QUAN-ZHONG LI, ZHI-GANG ZHAO

**Affiliations:** Department of Endocrinology, The People’s Hospital of Zhengzhou University (Henan Provincial People’s Hospital), Zhengzhou, Henan 450003, P.R. China

**Keywords:** type II diabetes mellitus, vildagliptin, α-glucosidase inhibitor, body weight, blood lipid, hepatorenal function

## Abstract

The aim of this study was to assess the efficacy of a combination therapy of vildagliptin plus an α-glucosidase inhibitor for patients with type II diabetes mellitus. Type II diabetic patients exhibiting poor glycemic control following α-glucosidase inhibitor treatment for at least two months were selected and randomly distributed into vildagliptin and placebo groups. The body weight, fasting blood glucose (FBG), postprandial glucose (PPG), glycated hemoglobin (HBA1c) and blood lipid levels and hepatorenal functions of the patients were determined before and 12 weeks after the trial. Following the trial, the FBG, PPG, HbA1c, cholesterol (CHOL) and triglyceride (TG) levels in the vildagliptin group were significantly decreased compared with the pretreatment levels (P<0.05), whereas only the PPG level in the placebo group decreased (P<0.05). The FBG, PPG and HbA1c levels in the vildagliptin group were markedly lower than those in the placebo group 12 weeks after the trial. A comparison of the body weights and hepatorenal functions before and after the trial or between groups did not show statistically significant differences. The combination therapy of vildagliptin plus an α-glucosidase inhibitor effectively reduced the FBG, PPG and HbA1c levels in patients without inducing weight gain or hepatorenal dysfunction. However, the therapy may have caused a reduction in the blood lipid levels.

## Introduction

The incidence of type II diabetes mellitus is gradually increasing. In addition to the extended course and development of diabetes, β-cell dysfunction is aggravated in diabetic patients. Thus, diet and an exercise program or intake of oral hypoglycemic drug alone fails to control blood glucose levels. As a result, a combination of drugs is frequently required to ensure controlled blood glucose and glycated hemoglobin (HBA1c) levels (1).

Dipeptidyl peptidase-4 (DPP-4) is a new target in the treatment of type II diabetes mellitus. Vildagliptin is a highly effective substrate-like DPP-4 inhibitor of the degradation of glucagon-like peptide-1 (GLP-1) and glucose-dependent insulin-like peptide (GIP) (2). α-glucosidase inhibitors may promote GLP-1 synthesis (3–7). Therefore, the combined use of vildagliptin and an α-glucosidase inhibitor may enhance the treatment efficacy and thus requires further study.

A 12-week randomized controlled trial was performed in 520 patients with type II diabetes who exhibited poor glycemic control [HbA1c >6.5%, fasting blood glucose (FBG) >7.0 mmol/l] following α-glucosidase inhibitor treatment between January 2012 and November 2012. The patients were randomly divided into vildagliptin and placebo groups, both of which continued treatment with the α-glucosidase inhibitor. The effects of a combination therapy of vildagliptin plus an α-glucosidase inhibitor on the fasting blood glucose (FBG), postprandial glucose (PPG) and HbA1c levels, body weight, blood lipid levels and hepatorenal functions of the patients were assessed.

## Materials and methods

### Subjects

A total of 520 patients (293 males and 227 females) were involved in the trial according to the following criteria: i) diagnosed with type II diabetes via a 75 g oral glucose tolerance test (based on the diagnostic criteria for diabetes mellitus issued by the American Diabetes Association in 2003); ii) aged 18–70 years old; iii) treated with metformin and an α-glucosidase inhibitor for more than two months; iv) with HbA1c >6.5% and FBG >7.0 mmol/l; v) exhibited hepatorenal function; vi) volunteered to participate in this study and signed an informed consent form. However, if patients met the following conditions they were rejected from the trial: i) has type I diabetes or other special diabetes; ii) younger than 18 years or older than 70 years; iii) had used hypoglycemic agents other than metformin and an α-glucosidase inhibitor; iv) has diabetic ketoacidosis, hyperglycemic nonketotic hyperosmolar syndrome or chronic complication that requires insulin treatment or must be treated with insulin under stress; v) showed detectable liver or kidney disease [with an alanine aminotransferase (ALT) or aspartate aminotransferase (AST) level greater than three times the upper limit of normal, or the total bilirubin (TBIL) is higher than one and half times the upper limit of normal, or CREA (creatinine) >115 μmol/l]; vi) pregnant or lactating women; vii) showed poor compliance. This study was conducted in accordance with the Declaration of Helsinki and was conducted with approval from the Ethics Committee of Henan Provincial People’s Hospital (Zhengzhou, China). Written informed consent was obtained from all participants. The participants received individual dietary guidance from an endocrinologist.

### Drug treatment and diet design

The patients randomly received vildagliptin (100 mg/day, orally in two separate doses) or a placebo according to the prescription. During the study, the patients self-monitored and recorded fingerstick blood glucose and hypoglycemic events. Twelve weeks after the trial, the body weight, FBG, PPG, HBA1c and blood lipid levels and hepatorenal functions of the patients were determined. The drugs were supplied by Henan Pharmaceuticals Co., Ltd. (Zhengzhou, China). α-glucosidase inhibitor was produced and manufactured by Bayer Healthcare Co., Ltd., (Beijing, China). Patients who forgot or adjusted the drug doses or treatment were excluded from the trial. α-glucosidase inhibitor was produced and manufactured by Bayer Healthcare Co., Ltd., (Office building of Beijing Fortune Center, Beijing, China).

### Statistical analysis

Data are presented as the mean ± standard deviation (SD). The Student’s t-test was used to compare between groups before and after the trial. Comparison of the associated indicators before and after the trial was performed using a paired Student’s t-test. The data were analyzed using SPSS software, version 13.0 (SPSS, Inc., Chicago, IL, USA). P<0.05 was considered to indicate a statistically significant result.

## Results

### Clinical characteristics

A total of 520 patients (293 males and 227 females), with 260 in the vildagliptin group and 260 in placebo group, were included in the trial. The patients were 49.16±14.036 (18–70) years of age, with body weights of 66.90±10.160 (45–80) kg. The patients had FBG levels of 9.09±1.618 (7.0–12.0) mmol/l, mean PPG (MPPG) levels of 10.43±1.531 (7.0–13.0) mmol/l and HbA1c levels of 8.80±1.602 (6.5–11.6)%. Twelve patients withdrew from the trial: in the vildagliptin group, three violated the treatment, two were lost to follow-up, and three showed an adverse event; in the placebo group, one violated the treatment, two were lost to follow-up, and one showed an adverse event. In total, 252 and 256 patients completed the clinical trial in the vildagliptin and placebo groups, respectively.

Table I shows no statistically significant differences between the age, body weight, FBG, MPPG, HbA1c, blood lipid or hepatorenal functions of the two groups (P>0.05).

### Comparison of associated indicators

The patients were rechecked 12 weeks after the trial. In the vildagliptin group, the FBG, MPPG, HbA1c, cholesterol (CHOL) and triglyceride (TG) levels significantly decreased (P<0.05), whereas the body weight and the transaminase and serum creatinine levels did not significantly change after the trial compared with those before the trial (P>0.05). In the placebo group, PPG decreased after the trial (P<0.05), but no statistical differences were found in the other indicators before and after the trial (Table II).

### Comparison of efficacy

Table III shows that twelve weeks after the trial, the FBG (P<0.0001), PPG (P<0.0001) and HbA1c (P=0.001) levels in the vildagliptin group were considerably lower than those of the placebo group, whereas the body weights, blood lipid levels and hepatorenal functions in the two groups showed no statistically significant differences between the groups.

## Discussion

Vildagliptin is a highly selective DPP-4 inhibitor that controls blood glucose by enhancing the response of islet α and β cells to glucose (8). Vildagliptin binds to DPP-4 and forms a DPP-4 complex to inhibit DPP-4 activity, increase the levels of active GLP-1 and GIP, promote insulin secretion by pancreatic β cells, and reduce glucagon secretion by α cells (9). GLP-1 likely prevents the deterioration of diabetes and reduces the risk of weight gain by controlling the appetite to compensate for the negative effects of existing antihyperglycemic agents. However, this peptide is readily hydrolyzed and inactivated by DPP-4 in the body and has a half-life of only two minutes. Consequently, DPP-4 inhibitors have become the research focus in the development of alternative antihyperglycemic agents.

Vildagliptin alone or combined with oral hypoglycemic drugs or insulin has been demonstrated in numerous randomized controlled clinical trials to effectively reduce the FBG and HbA1c levels in patients with type II diabetes (10–15). However, to the best of our knowledge, the efficacy of a combination therapy of vildagliptin plus an α-glucosidase inhibitor has not been reported. Therefore, the clinical trial described in the present study aimed to compare the efficacies of vildagliptin and placebo in patients with poor glycemic control following α-glucosidase inhibitor treatment alone and observe any adverse effects of vildagliptin. In the vildagliptin group, two cases of hypoglycemia were observed, as well as one case of diarrhea that disappeared after three days and was likely not associated with vildagliptin. In the placebo group, one case of hypoglycemia was recorded. Eight patients withdrew from the trial in the vildagliptin group, whereas four patients withdrew from the trial in the placebo group.

The present study revealed that vildagliptin significantly reduced the FBG, PPG and HbA1c levels in patients compared with those prior to the vildagliptin treatment and those following the placebo treatment. This result indicates that vildagliptin is able to control FBG and PPG levels. A previous study (8) has shown that vildagliptin alone reduces HbA1c by 0.5–1.0%. This result slightly differs from our data due to the combined use of vildagliptin and an α-glucosidase inhibitor in the present study. Following treatment, the weight slightly decreased in the vildagliptin group; however, the difference from the pretreatment levels was not statistically significant. In the vildagliptin group, the CHOL and TG levels also significantly decreased following treatment.

A meta-analysis of the results of 38 phase II/III clinical studies suggested the absence of a correlation between vildagliptin and increased risk of liver events or elevated hepatase (16). One retrospective study of a DPP-4 inhibitor demonstrated that DPP-4 inhibitors are correlated with reduced total cholesterol levels (17). These findings are consistent with the results of the present study. The present clinical trial also showed that vildagliptin induced reductions in CHOL and TG levels.

Previous studies (18–20) have shown that numerous treatments for type II diabetes cause body weight to increase. However, the present study found no significant changes in body weight following the vildagliptin treatment. These results indicate that the risk of weight gain during vildagliptin treatment is low.

The combination of the DPP-4 inhibitor vildagliptin and an α-glycosidase inhibitor appears feasible. DPP-4 inhibitors function by inhibiting the degradation of GLP-I and GIP (2), whereas α-glycosidase inhibitors may promote the secretion of GLP-I (3–7). The combination of these two inhibitors is likely to increase the activity of GLP-I in reducing blood glucose levels. Several *in vitro* animal studies and clinical trials have shown that DPP-4 inhibitors are able to stimulate the proliferation and differentiation of pancreatic β cells, increase the number of β cells and inhibit the apoptosis of β cells (21). These findings indicate that vildagliptin improves the functioning of pancreatic β cells. The current results may increase the acceptability of the combined treatment to patients with diabetes.

In conclusion, the combination therapy of vildagliptin plus an α-glucosidase inhibitor effectively reduced the FBG, PPG and HbA1c levels and possibly decreased the blood lipid levels in patients with type II diabetes without disrupting the hepatorenal function or inducing weight gain or hypoglycemia. Therefore, in terms of safety and efficacy, the combined use of vildagliptin and an α-glucosidase inhibitor is considered an effective hypoglycemic therapy for type II diabetic patients.

## Figures and Tables

**Table I:** Clinical characteristics of the subjects.

Variables	Vildagliptin group (mean ± SD)	Placebo group (mean ± SD)	t-value	P-value
Age (years)	48.65±15.42	49.67±12.65	−0.23	0.84
Height (cm)	166.50±6.46	164.61±6.45	0.89	0.39
Weight (kg)	66.14±10.42	67.67±9.90	0.44	0.67
FBG (mmol/l)	9.32±1.69	8.86±1.55	0.85	0.40
MPPG (mmol/l)	10.44±1.57	10.43±1.50	0.04	0.98
HbA1c (%)	8.96±1.82	8.65±1.39	0.44	0.66
ALT (U/l)	30.44±15.85	23.28±9.93	1.63	0.11
AST (U/l)	22.89±7.68	20.67±5.32	1.22	0.32
CHOL (mmol/l)	4.98±1.01	4.63±1.10	1.01	0.34
TG (mmol/l)	1.95±1.39	1.95±1.34	0.19	0.85
HDL (mmol/l)	1.39±0.31	1.05±0.28	1.41	0.17
LDL (mmol/l)	2.87±0.66	2.55±0.83	1.31	0.20
Urea (mmol/l)	5.58±1.65	5.67±1.27	−0.20	0.85
CREA (μmol/l)	57.28±13.57	60.67±20.36	−0.59	0.56

FBG, fasting blood glucose; MPPG, postprandial glucose; HbA1c, glycated hemoglobin; ALT, alanine aminotransferase; AST, aspartate aminotransferase; CHOL, total cholesterol; TG, triglycerides; HDL, high-density lipoproteins; LDL, low-density lipoproteins; CREA, creatinine; SD, standard deviation.

**Table II:** Comparison of associated indicators before and after vildagliptin or placebo treatments.

Variables	Before treatment (mean ± SD)	After treatment (mean ± SD)	t-value	P-value
Vildagliptin group (n=252)				
Weight (kg)	66.14±10.42	65.94±10.55	0.06	0.96
FBG (mmol/l)	9.32±1.69	6.62±0.81	6.27	<0.0001
MPPG (mmol/l)	10.44±1.57	9.03±0.64	6.12	<0.0001
HbA1c (%)	8.96±1.82	6.65±1.44	4.25	<0.0001
ALT (U/l)	30.44±15.85	20.84±7.08	2.32	0.63
AST (U/l)	22.89±7.68	20.22±4.89	1.25	0.22
CHOL (mmol/l)	4.98±1.01	4.70±0.88	1.52	0.05
TG (mmol/l)	1.95±1.39	1.45±0.84	1.54	0.03
HDL (mmol/l)	1.39±0.31	1.15±0.24	0.39	0.70
LDL (mmol/l)	2.87±0.66	2.58±0.82	1.28	0.24
Urea (mmol/l)	5.58±1.65	5.81±2.10	−0.37	0.71
CREA (μmol/l)	57.28±13.57	57.11±14.56	0.06	0.96
Placebo group (n=256)				
Weight (kg)	67.67±9.90	66.69±8.45	−0.01	0.99
FBG (mmol/l)	8.86±1.55	8.60±1.24	0.58	0.58
MPPG (mmol/l)	10.43±1.50	9.70±1.36	1.54	0.04
HbA1c (%)	8.65±1.39	8.23±1.18	1.06	0.30
ALT (U/l)	23.28±9.93	26.89±8.61	−1.20	0.24
AST (U/l)	20.67±5.32	23.77±4.37	−1.88	0.07
CHOL (mmol/l)	4.63±1.10	4.67±0.95	−0.12	0.90
TG (mmol/l)	1.95±1.34	1.99±1.33	−0.10	0.92
HDL (mmol/l)	1.05±0.28	1.41±0.30	−1.89	0.05
LDL (mmol/l)	2.55±0.83	2.67±0.81	−0.47	0.57
Urea (mmol/l)	5.67±1.27	5.33±1.42	0.92	0.36
CREA (μmol/l)	60.67±20.36	67.61±21.95	−0.56	0.59

FBG, fasting blood glucose; MPPG, postprandial glucose; HbA1c, glycated hemoglobin; ALT, alanine aminotransferase; AST, aspartate aminotransferase; CHOL, total cholesterol; TG, triglycerides; HDL, high-density lipoproteins; LDL, low-density lipoproteins; CREA, creatinine; SD, standard deviation.

**Table III:** Comparison of important indicators between vildagliptin and placebo groups 12 weeks after the trial.

Variables	Vildagliptin group (n=252; mean ± SD)	Placebo group (n=256; mean ± SD)	t-value	P-value
Weight (kg)	65.94±10.55	66.69±8.45	0.42	0.21
FBG (mmol/l)	6.62±0.81	8.60±1.24	−6.16	<0.0001
MPPG (mmol/l)	9.03±0.64	9.70±1.36	−4.89	<0.0001
HbA1c (%)	6.65±1.44	8.23±1.18	−4.21	0.001
ALT (U/l)	20.84±7.08	26.89±8.61	−3.32	0.14
AST (U/l)	20.22±4.89	23.77±4.37	−2.24	0.08
CHOL (mmol/l)	4.70±0.88	4.67±0.95	−0.66	0.59
TG (mmol/l)	1.45±0.84	1.99±1.33	−1.80	0.10
HDL (mmol/l)	1.15±0.24	1.41±0.30	−0.99	0.43
LDL (mmol/l)	2.58±0.82	2.67±0.81	−0.47	0.72
Urea (mmol/l)	5.81±2.10	5.33±1.42	0.93	0.35
CREA (μmol/l)	57.11±14.56	67.61±21.95	−1.90	0.24

FBG, fasting blood glucose; MPPG, postprandial glucose; HbA1c, glycated hemoglobin; ALT, alanine aminotransferase; AST, aspartate aminotransferase; CHOL, total cholesterol; TG, triglycerides; HDL, high-density lipoproteins; LDL, low-density lipoproteins; CREA, creatinine; SD, standard deviation.

## References

[1] Samraj GP (2011). Vildagliptin for the treatment of diabetes. Therapy.

[2] Croxtall JD, Keam SJ (2008). Vildagliptin: a review of its use in the management of type 2 diabetes mellitus. Drugs.

[3] Masuda K, Aoki K, Terauchi Y (2011). Effects of miglitol taken just before or after breakfast on plasma glucose, serum insulin, glucagon and incretin levels after lunch in men with normal glucose tolerance, impaired fasting glucose or impaired glucose tolerance. J Diabet Invest.

[4] Aoki K, Kamiyama H, Yoshimura K, Shibuya M, Masuda K, Terauchi Y (2012). Miglitol administered before breakfast increased plasma active glucagon-like peptide-1 (GLP-1) levels after lunch in patients with type 2 diabetes treated with sitagliptin. Acta Diabetol.

[5] Narita T, Katsuura Y, Sato T (2009). Miglitol induces prolonged and enhanced glucagon-like peptide-1 and reduced gastric inhibitory polypeptide response after ingestion of a mixed meal in Japanese Type 2 diabetic patients. Diabet Med.

[6] Arakawa M, Ebato C, Mita T (2008). Miglitol suppresses the postprandial increases in interleukin 6 and enhances active glucagon-like peptide 1 secretion in viscerally obese subjects. Metabolism.

[7] Aoki K, Miyazaki T, Nagakura J, Orime K, Togashi Y, Terauchi Y (2010). Effects of pre-meal versus post-meal administration of miglitol on plasma glucagon-like peptide-1 and glucose dependent insulinotropic polypeptide levels in healthy men. Endocr J.

[8] Pi-Sunyer FX, Schweizer A, Mills D, Dejager S (2007). Efficacy and tolerability of vildagliptin monotherapy in drug-naïve patients with type 2 diabetes. Diabetes Res Clin Pract.

[9] Tahrani AA, Piya MK, Barnett AH (2009). Drug evaluation: vildagliptin-metformin single-tablet combination. Adv Ther.

[10] Ahrén B (2009). Clinical results of treating type 2 diabetic patients with sitagliptin, vildagliptin or saxagliptin - diabetes control and potential adverse events. Best Pract Res Clin Endocrinol Metab.

[11] Bosi E, Dotta F, Jia Y, Goodman M (2009). Vildagliptin plus metformin combination therapy provides superior glycaemic control to individual monotherapy in treatment-naive patients with type 2 diabetes mellitus. Diabetes Obes Metab.

[12] Devendra D, Gohel B, Bravis V (2009). Vildagliptin therapy and hypoglycaemia in Muslim type 2 diabetes patients during Ramadan. Int J Clin Pract.

[13] Matthews DR, Dejager S, Ahren B (2010). Vildagliptin add-on to metformin produces similar efficacy and reduced hypoglycaemic risk compared with gimepiride, with no weight gain: results from a 2-year study. Diabetes Obes Metab.

[14] Iwamoto Y, Kashiwagi A, Yamada N (2010). Efficacy and safety of vildagliptin and voglibose in Japanese patients with type 2 diabetes: a 12-week, randomized, double-blind, active-controlled study. Diabetes Obes Metab.

[15] Schweizer A, Dejager S, Foley JE, Shao Q, Kothny W (2011). Clinical experience with vildagliptin in the management of type 2 diabetes in a patient population ≥ 75 years: a pooled analysis from a data base of clinical trials. Diabetes Obes Metab.

[16] Ligueros-Saylan M, Foley JE, Schweizer A, Couturier A, Kothny W (2010). An assessment of adverse effects of vildagliptin versus comparators on the liver, the pancreas, the immune system, the skin and in patients with impaired renal function from a large pooled database of Phase II and III clinical trials. Diabetes Obes Metab.

[17] Richard KR, Shelburne JS, Kirk JK (2011). Tolerability of dipeptidyl peptidase-4 inhibitors: a review. Clin Ther.

[18] Kahn SE, Haffner SM, Heise MA (2006). Glycemic durability of rosiglitazone, metformin, or glyburide monotherapy. N Engl J Med.

[19] Dormandy JA, Charbonnel B, Eckland DJ (2005). Secondary prevention of macrovascular events in patients with type 2 diabetes in the PROactive study (PROspective pioglitAzone Clinical Trial In macroVascular Events): a randomised controlled trial. Lancet.

[20] Ilag LL, Kerr L, Malone JK, Tan MH (2007). Prandial premixed insulin analogue regimens versus basal insulin analogue regimens in the management of type 2 diabetes: an evidence-based comparison. Clin Ther.

[21] Neumiller JJ (2009). Differential chemistry (structure), mechanisnm faction, and pharmacology of GLP-1 receptor agonists and DPP-4 inhibitor. J Am Pharm Assoc (2003).

